# tRNA shape is an identity element for an archaeal pyrrolysyl-tRNA synthetase from the human gut

**DOI:** 10.1093/nar/gkad1188

**Published:** 2023-12-15

**Authors:** Natalie Krahn, Jingji Zhang, Sergey V Melnikov, Jeffery M Tharp, Alessandra Villa, Armaan Patel, Rebecca J Howard, Haben Gabir, Trushar R Patel, Jörg Stetefeld, Joseph Puglisi, Dieter Söll

**Affiliations:** Department of Molecular Biophysics and Biochemistry, Yale University, New Haven, CT 06520, USA; Department of Structural Biology, Stanford University School of Medicine, Stanford, CA 94305, USA; Biosciences Institute, Newcastle University, Newcastle upon Tyne, NE2 4HH, UK; Department of Molecular Biophysics and Biochemistry, Yale University, New Haven, CT 06520, USA; PDC-Center for High Performance Computing, KTH-Royal Institute of Technology, Stockholm, SE-100 44, Sweden; Department of Molecular Biophysics and Biochemistry, Yale University, New Haven, CT 06520, USA; Department of Biochemistry and Biophysics, Science for Life Laboratory, Stockholm University, Solna, SE-171 65, Sweden; Department of Chemistry, University of Manitoba, Winnipeg, MB R3T 2N2, Canada; Department of Chemistry and Biochemistry, Alberta RNA Research and Training Institute, University of Lethbridge, Lethbridge, AB T1K 2E1, Canada; Li Ka Shing Institute of Virology, University of Alberta, Edmonton, AB T6G 2E1, Canada; Department of Microbiology, Immunology & Infectious Diseases, Cumming School of Medicine, University of Calgary, Calgary, AB T2N 4N1, Canada; Department of Chemistry, University of Manitoba, Winnipeg, MB R3T 2N2, Canada; Department of Microbiology, University of Manitoba, Winnipeg, MB R3T 2N2, Canada; Department of Structural Biology, Stanford University School of Medicine, Stanford, CA 94305, USA; Department of Molecular Biophysics and Biochemistry, Yale University, New Haven, CT 06520, USA; Department of Chemistry, Yale University, New Haven, CT 06520, USA

## Abstract

Protein translation is orchestrated through tRNA aminoacylation and ribosomal elongation. Among the highly conserved structure of tRNAs, they have distinguishing features which promote interaction with their cognate aminoacyl tRNA synthetase (aaRS). These key features are referred to as identity elements. In our study, we investigated the tRNA:aaRS pair that installs the 22^nd^ amino acid, pyrrolysine (tRNA^Pyl^:PylRS). Pyrrolysyl-tRNA synthetases (PylRSs) are naturally encoded in some archaeal and bacterial genomes to acylate tRNA^Pyl^ with pyrrolysine. Their large amino acid binding pocket and poor recognition of the tRNA anticodon have been instrumental in incorporating >200 noncanonical amino acids. PylRS enzymes can be divided into three classes based on their genomic structure. Two classes contain both an N-terminal and C-terminal domain, however the third class (ΔpylSn) lacks the N-terminal domain. In this study we explored the tRNA identity elements for a ΔpylSn tRNA^Pyl^ from *Candidatus Methanomethylophilus alvus* which drives the orthogonality seen with its cognate PylRS (MaPylRS). From aminoacylation and translation assays we identified five key elements in ΔpylSn tRNA^Pyl^ necessary for MaPylRS activity. The absence of a base (position 8) and a G–U wobble pair (G28:U42) were found to affect the high-resolution structure of the tRNA, while molecular dynamic simulations led us to acknowledge the rigidity imparted from the G–C base pairs (G3:C70 and G5:C68).

## Graphical Abstract

**Graphical Abstract:**
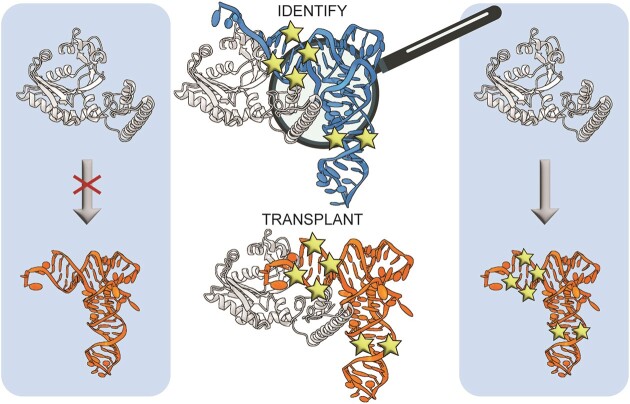

## Introduction

Transfer RNAs (tRNAs), facilitate the fundamental process of protein translation, converting an mRNA sequence into protein. Most tRNAs involved in protein translation have a highly conserved tertiary structure to interact with the elongation factor, EF-Tu in bacteria, and efficiently pass through the ribosome. However, there are distinguishing features of each tRNA which are recognized by their cognate aminoacyl tRNA synthetase (aaRS) for attachment of the corresponding amino acid onto the 3′CCA end of the tRNA. These are referred to as tRNA identity elements and include 4–7 bases that either promote interaction with its cognate aaRS (determinant) or prevent interaction with non-cognate aaRSs (anti-determinant). Identity elements have been well-characterized for the 20 canonical tRNA isoacceptor groups in specific organisms (1,2) and recently expanded to tRNA genes from all domains of life (3).

**Figure 1.**
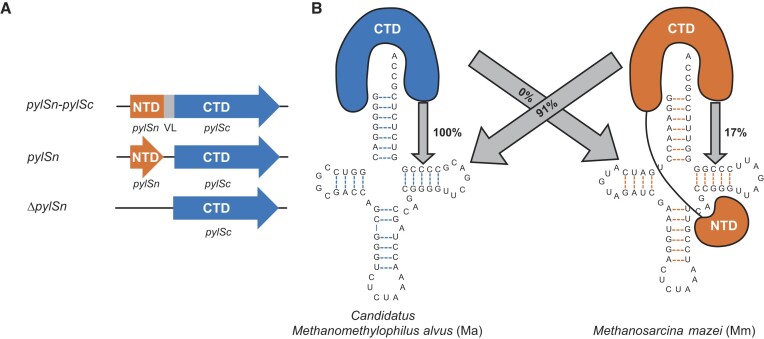
(**A**) Schematic of domains for the three different classes of PylRS enzymes (NTD, N-terminal domain; VL, variable linker; CTD, C-terminal domain). (**B**) Cloverleaf structures of *Ca. M. alvus* and *M. mazei* tRNA^Pyl^ (Ma and Mm, respectively) and depiction of the domains present in their cognate PylRS (MaPylRS and MmPylRS, respectively). Image representation is adapted from (47). MaPylRS is of the Δ*pylSn* class and MmPylRS is of the *pylSn* class. The numbers in the arrows represent the relative translation activity of the PylRS towards that tRNA^Pyl^ by our *in vivo* assays with Pyl as a substrate.

In some organisms there are additional translation systems which repurpose stop codons to facilitate insertion of up to 22 amino acids. There is evidence of UGA recoding in all three domains of life to insert selenocysteine (the 21st amino acid), while UAG recoding has been found mostly in archaeal species, and some bacteria, to insert the 22nd amino acid, pyrrolysine (Pyl) (4,5). Similar to the 20 canonical amino acids, Pyl also has a dedicated tRNA:aaRS pair (tRNA^Pyl^:PylRS) for protein translation. Three different classes of PylRS enzymes have been identified, named according to the domains present and their genomic organization. The *pylSc-pylSn* class (found in *Methanosarcina mazei*) has the C-terminal domain (CTD) and N-terminal domain (NTD) fused together by a linker, while in *pylSn* (found in *Desulfitobacterium hafniense*) the CTD and NTD are two separate genes that come together after translation (Figure 1A). The last class, *ΔpylSn* (found in *Candidatus Methanomethylophilus alvus*), is devoid of the tRNA-binding NTD, yet is found to be highly active in *Escherichia coli* (Figure 1) (6). This is striking given that the NTD is required for efficient *in vivo* activity of *pylSn* and *pylSn-pylSc* classes of PylRS (7).

The *ΔpylSn* class enzymes are orthogonal to the other two classes of PylRS, unable to recognize their respective tRNA^Pyl^ (Figure 1B) (8). This suggests that there are identity elements on tRNAs from the *ΔpylSn* class (ΔN tRNA^Pyl^) that facilitate aminoacylation by a CTD-only PylRS. The 3 nt variable arm of tRNA^Pyl^ has been shown to be important for aminoacylation by *pylSn* and *pylSn-pylSc* class enzymes (8,9). A variable arm that is longer (4 nt or more), sterically clashes with the NTD of PylRS, preventing recognition of this enzyme to any other endogenous tRNA (9). PylRS from the *ΔpylSn* class, however, does not have the NTD, and therefore this feature is not considered an identity element. Instead, by adding in an extra nucleotide to the variable arm of a ΔN tRNA^Pyl^, this creates an orthogonal translation system between PylRS enzymes from the other two classes (8).

Here, we systematically investigated which nucleotides were driving the interaction between PylRS and tRNA^Pyl^ from *M. alvus* (ΔN tRNA^Pyl^). Aminoacylation and translation assays found five base positions (including the absence of a base), that are important for activity with *M. alvus* PylRS (MaPylRS). Structural studies revealed that two of the five base positions induced changes in the tRNA structure for *M. alvus* tRNA^Pyl^ (Ma tRNA^Pyl^) when compared to *M. mazei* tRNA^Pyl^ (Mm tRNA^Pyl^). This left three other base positions with no clarity on their importance to facilitate recognition by MaPylRS. Through molecular dynamic (MD) simulations of our tRNA^Pyl^ variants, we realized that all five base positions we identified, work together to create a defined structure recognized by MaPylRS. Our data shows for the first time that the absence of a base provides a new perspective on tRNA identity and the mechanism of enzyme recognition may have a future in personalized medicine with tRNA therapeutics.

## Materials and methods

### Preparation of materials for analysis

#### pBAD_sfGFP2am_tRNA

tRNA variants were cloned into pBAD_sfGFP2am (10,11) in the reverse direction (3′ to 5′). First the pBAD_sfGFP2am vector was opened through amplification of the plasmid around the tRNA. Following DpnI (New England Biolabs®) digest and cleanup to remove the methylated parent plasmid, the product was used as a template to insert the tRNA via two overlapping primers (W.M. Keck Foundation, Yale University). The first primer, in the reverse direction (3′ to 5′), had at least 15 bp complimentary to the rrcn terminator of pBAD_sfGFP2am and a tail containing a portion of the tRNA sequence (∼50 bp). The second primer, in the forward direction (5′ to 3′), had at least 20 bp complimentary to the 3′ end of the first primer, continued with the rest of the tRNA sequence, and then ended with at least 15 bp complimentary to the Lpp promoter of pBAD_sfGFP2am. NEBuilder HiFi (New England Biolabs®) was used to ensure complete vector assembly before transformation into DH5α cells for plasmid prep and sequencing (W.M. Keck Foundation, Yale University).

#### pMW-PylRS-pylBCD

The *pylBCD* variant 3f2 for improved Pyl biosynthesis was amplified out of its parent plasmid (a gift from Dr Joanne M.L. Ho) (12) with the addition of an EM7 promoter. The pMW-PylRS plasmids (11) containing enzymes from *Ca. M. alvus*, *M. mazei* or devoid of an enzyme were opened downstream of the kanamycin resistance gene though amplification of primers in the opposite direction. The *pylBCD* cassette was inserted into the opened pMW-PylRS with NEBuilder HiFi (New England Biolabs®) due to complimentary regions inserted with PCR amplification.

#### MaPylRS protein expression and purification

*Escherichia coli* BL21(DE3) were transformed with a pET28a vector contain N-terminally His-tagged MaPylRS. Transformed cells were grown in 2× YT media supplemented with 50 μg/ml kanamycin at 37°C, 250 rpm, to OD_600_ = 0.6 at which point the temperature was lowered to 25°C and protein expression was induced with 0.1 mM isopropyl-β-d-thiogalactoside (IPTG). After 18 h, cells were harvested via centrifugation, resuspended in 50 mM Tris pH 8.0, 50 mM NaCl, 5 mM MgCl_2_ (Buffer A) and lysed by sonication. The cell debris was removed via centrifugation and proteins were precipitated from the clarified lysate in two fractions using 30%, followed by 60% (w/v) ammonium sulfate. Precipitated proteins from the 60% (w/v) ammonium sulfate fraction were redissolved in Buffer B (50 mM Tris pH 8.0, 250 mM NaCl, 5 mM MgCl_2_) containing 20 mM imidazole and purified using Ni-NTA resin in a gravity flow column. Bound proteins were eluted using Buffer B containing 250 mM imidazole, and then the buffer exchanged to Buffer A using an Amicon centrifugal filter unit (10 kDa MWCO). The protein was further purified on a Q Sepharose Fast Flow column, eluting with Buffer B, followed by a Superdex Increase 10/300 GL size exclusion column pre-equilibrated with storage buffer (20 mM Tris pH 8.0, 200 mM NaCl, 5 mM MgCl_2_, 1 mM dithiothreitol). Pure fractions were concentrated to >30 mg/ml and flash frozen in liquid nitrogen.

#### In vitro transcription of tRNAs

DNA template for *in vitro* transcription of the tRNA^Pyl^ variants was amplified from the appropriate pBAD_sfGFP2am_pylT plasmid. The forward primer contained a 14 bp tail at the 5′ end, the T7 promoter sequence, and 16 bp complimentary to the 5′ end of the tRNA sequence. The reverse primer was complimentary to the 3′ end of the tRNA. PCR amplification was done using GoTaq® Master Mix (Promega) and amplified DNA was purified with standard phenol–chloroform extraction. Transcription reactions were set up as described previously (13) using 18–22 mM MgCl_2_ and purified.

### Aminoacylation and translation studies

#### In vitro aminoacylation assays

The *in vitro* transcribed tRNA^Pyl^ variants were first labelled at the 3′ end with [α-^32^P]-ATP, as described previously (14). Aminoacylation reactions were set up with 5 μM of ^32^P-labelled tRNA^Pyl^, 625 nM MaPylRS, and 250 μM Pyl in buffer (100 mM HEPES [pH 7.2], 100 mM NaCl, 25 mM MgCl_2_, 5 mM ATP, 1 mM dithiothreitol). Reactions were quenched for 30 min in 5 μl solution (0.5 U P1 nuclease in 200 mM sodium acetate, pH 5.5) at time points 15 s, 1 min and 15 min. Aminoacylation efficiency was monitored in these reactions by separation of charged from uncharged tRNA on a TLC plate, as described previously (14). Two-sided *t*-tests were performed for all activity assays with *n* = 3.

#### In vivo fluorescence assays

DH10B cells were transformed with pBAD_sfGFP2am_pylT (containing the corresponding tRNA^Pyl^ variant) and pMW_PylRS_pylBCD (with the PylRS of interest) and grown overnight on selection plates. Single colonies were transferred to 150 μl expression media (Luria Broth containing 100 μg/ml ampicillin, 50 μg/ml kanamycin, 0.1% (w/v) arabinose, and 1 mM IPTG) in a 96-well black plate with clear bottom. Plates were incubated at 37°C for 24 h in a Synergy HTX Plate reader (BioTek). Fluorescence (Ex. 485 nm, Em. 528 nm) and *A*_600_ measurements were taken every 15 min with a minimum of four biological replicates. Relative activity of translation was calculated at 16 h by dividing the fluorescent measurement by the *A*_600_ reading and comparing its activity to MaPylRS with Ma tRNA^Pyl^. Two-sided *t*-tests were performed for all activity assays with *n* = 4.

### Structural studies on the ribosome

#### Sample preparation

Cryo-EM specimens of Ma tRNA^Pyl^ and Mm tRNA^Pyl^ were prepared as described previously (13). The beam-induced motion of the sample and the instability of the stage due to thermal drift was corrected using MotionCor2 (15). The contrast transfer function (CTF) of each micrograph was estimated using CTFFIND4 (16). Imaged particles were picked using the Autopicker algorithm included in the RELION software (17).

#### Cryo-EM classification

For Ma tRNA^Pyl^ in complex with the ribosome (Supplementary Figure S1), 238 6471 particles were picked from 14 092 micrographs. 2D classification of the four-times binned particles were used to separate ribosome-like particles from ice-like and/or debris-like particles picked by the Autopicker algorithm. 1 203 474 particles were saved for 3D classification. Of those, 920 880 particles were selected from high-resolution 3D classes for focused 3D classification. Focused 3D classification was performed on re-extracted particles without binning. 412 295 particles with Ma tRNA^Pyl^ density were selected for auto-refinement.

For Mm tRNA^Pyl^ in complex with the ribosome (Supplementary Figure S2), 5 385 559 particles were picked from 27 651 micrographs. 2D classification of the four-times binned particles were used to separate ribosome-like particles from ice-like and/or debris-like particles picked by the Autopicker algorithm. 3 240 791 particles were saved for 3D classification. Of those, 3 166 597 particles were selected from high-resolution 3D classes for focused 3D classification. Focused 3D classification was performed on re-extracted particles without binning. 405 164 particles with Mm tRNA^Pyl^ density were selected for auto-refinement.

Un-binned particles from these classes were subjected to auto-refinement. The final density map was sharpened by applying a negative B-factor estimated by automated procedures. Local resolution variations were estimated using ResMap (18) and visualized with UCSF Chimera (19).

#### Model building and refinement

Models of the *E. coli* 70S ribosome (5WE4) were docked into the maps using UCSF Chimera (19). All models were manually adjusted, and then de-novo built for the missing residues in Coot (20) followed by Phenix (21) and Refmac (22) refinement. All figures showing electron densities and atomic models were created using UCSF Chimera (19) and PyMOL Molecular Graphics Systems.

#### Molecular dynamic simulations

The A site cryo-EM tRNA structures of both Ma tRNA^Pyl^ (PDBID:8UPT) and Mm tRNA^Pyl^ (PDBID:8UPY) were removed from the ribosome for simulation in solution. tRNA^Pyl^ variants were modified in PyMOL prior to simulation and Dh tRNA^Pyl^ (PDBID:2ZNI) monomer was extracted from its crystal structure. For all tRNAs, nine Mg ions were added to their structures by superimposing the tRNA backbone with tRNA^Phe^ (PDBID:1EHZ) and extracting the Mg ion's position from that structure. Each tRNA was solvated in a dodecahedron-shaped box (having a distance of at least 1 nm from the box edge) with TIP3P water molecules (23) and neutralized with 0.15 M NaCl. The nucleic acid structure and ions were described using the CHARMM36 force field (24). All simulations were performed using the GROMACS (version 2021.4) package. To maintain constant temperature and pressure, a V-rescale and C-rescale coupling were applied, respectively. Each tRNA was equilibrated to a temperature of 300 K with a coupling time of 0.1 ps and pressure of 1 bar with a coupling time of 1.0 ps. The isothermal compressibility was 4.5 × 10^−5^ bar^−1^. A leap-frog integrator with an integration time step of 0.002 fs was used. The H-bonds were constrained using P-LINCS algorithm (25) with a short-range electrostatic and van der Waals cutoff of 1.2 nm. Three independent replicas were performed for each molecular system to avoid simulations from being stuck in a local minimum. Simulations were run for 100 ns each which allowed for convergence. For analysis purposes, the three trajectories have been combined. From this an average structure was generated and root mean squared fluctuations (RMSF) were calculated for each base.

### Biophysical analysis of MaPylRS

#### Solution X-ray scattering

Solution X-ray scattering data for MaPylRS was collected at 5 mg/ml in buffer (50 mM MES, pH 5.2, 500 mM NaCl, 10 mM MgCl_2_) at Diamond Light Source (Didcot, UK) using HPLC-SAXS setup as performed previously (26). A sample volume of 50 μl was injected into an Agilent 1200 (Agilent Technologies) HPLC connected to a specialized flow cell. We used a Shodex KW402.5-4F column to remove any possible aggregated/degraded material. Each size-exclusion frame was exposed to X-rays for 3 s, followed by SAXS data analysis. Data minimization was first performed to buffer subtract peak regions and then data was merged using the ScÅtter program. The merged data was further analysed using Guinier approximation to obtain the radius of gyration (*R*_g_) and determine the homogeneity of the samples. We also performed Dimensionless Kratky analysis (27) to determine if the samples were folded. Next, we utilized the GNOM program (28) to obtain the pair-distance distribution (*P*(*r*)), which provided the *R_g_* and the maximum particle dimension (*D*_max_). Subsequently, the *P*(*r*) information was used to calculate 12 low-resolution *ab initio* shape envelopes for MaPylRS with the program DAMMIN (29), as described previously (30,31). All 12 models were averaged and filtered to obtain a representative model through DAMAVER (32).

#### Sedimentation velocity

Sedimentation velocity analytical ultracentrifugation (SV-AUC) was conducted on PylRS samples prepared at concentrations of 0.3, 0.6 and 1.2 mg/ml in 50 mM MES, pH 5.2, 500 mM NaCl, 10 mM MgCl_2_. The SV-AUC experiments were carried out using a ProteomeLab™ XL-I ultracentrifuge (Beckman Coulter Inc.) and an An50Ti 8-cell rotor (Beckman Coulter Inc.). Standard 12 mm Epon double-sector cells were filled with 420 μl of MaPylRS on one side and 420 μl of buffer (50 mM MES, pH 5.2, 500 mM NaCl, 10 mM MgCl_2_) on the other side. Following an overnight equilibration period at 20°C under vacuum, the samples were subjected to sedimentation for a duration of 24 h at 30 000 rpm, collecting intensity data at 288 nm and interference data. Experimental data were processed with UltraScan-III (33) as previously described (34).

#### Dynamic light scattering

Hydrodynamic radius (*R*_h_) distributions of MaPylRS were obtained using a Nano-S Zetasizer (Malvern) instrument as described previously (31). Five measurements were obtained for concentrations between 0.20 and 1.60 mg/ml and averaged distributions plotted.

## Results

### Identification of tRNA^Pyl^ elements required for MaPylRS activity

**Figure 2.**
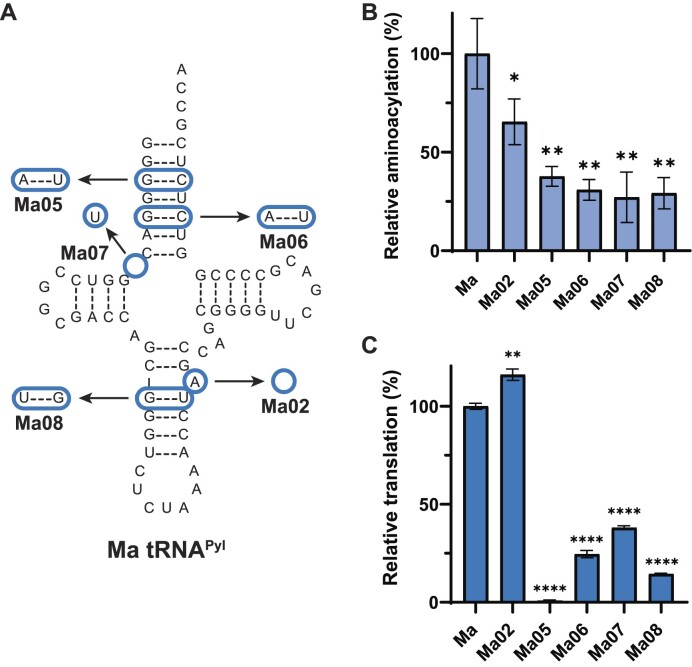
Identification of elements required for MaPylRS to recognize Ma tRNA^Pyl^. (**A**) Ma tRNA^Pyl^ variant cloverleaf structures. Bases that are changed or missing are represented with a blue circle. All variants include the absence of A42a as shown with Ma02. (**B**) *In vitro* assays with Ma tRNA^Pyl^ variants show the effect of tRNA mutations on aminoacylation efficiency with MaPylRS. Data represent the average of at least three biological replicates with the error bars corresponding to the standard deviation. (**C**) *In vivo* fluorescent readthrough assays with Ma tRNA^Pyl^ variants show the effect of tRNA mutations on translation with MaPylRS. Statistical analysis performed with a paired *t*-test and significance represented as stars.

To determine the key elements of Ma tRNA^Pyl^ that are responsible for its interaction and subsequent aminoacylation by MaPylRS, we systematically engineered mutations into the Ma tRNA^Pyl^ sequence. We compared the tRNA sequence of Ma tRNA^Pyl^, which can be aminoacylated with MaPylRS, to Mm tRNA^Pyl^ which cannot (Figure 1) (8). From this, we found the nucleotide differences which we decided to test. The first major difference we found when comparing the two tRNA sequences was the presence of an A-bulge in the anticodon stem of Ma tRNA^Pyl^. The A-bulge is found at position 42a, and is termed as such because this single nucleotide does not have a complementary base in the anticodon stem to base pair (Figure 2A). To confirm prior results that found this base to be unnecessary for function (35), we engineered Ma tRNA^Pyl^ variant Ma02 (Figure 2A). Removal of the A-bulge had no negative effect on the system, with *in vitro* aminoacylation reduced to 65% (*P* < 0.05) (Figure 2B) and *in vivo* readthrough of sfGFP increased to 116% (*P* < 0.01) (Figure 2C). Since Mm tRNA^Pyl^ does not have a bulge, we simplified the comparison between the two tRNAs, and the A-bulge was removed from Ma tRNA^Pyl^, giving rise to Ma02 as the scaffold for further mutagenesis.

Given the known interaction of *D. hafniense* PylRS with the acceptor stem of its cognate tRNA^Pyl^ (Dh) (36) we then focused on the acceptor stem of Ma tRNA^Pyl^. Compared to Mm tRNA^Pyl^, the acceptor stem of Ma tRNA^Pyl^ is more G:C rich. Therefore, we chose to individually mutate the two G:C base pairs in the acceptor stem (G3:C70 and G5:C68) to A:U (found in Mm tRNA^Pyl^), generating variants Ma05 and Ma06. Both mutations induced a significant decrease in *in vitro* (*P* < 0.01, Figure 2B) and *in vivo* (*P* < 0.0001, Figure 2C) activity. We continued past the acceptor stem and identified that Ma tRNA^Pyl^ is missing a base in the connecting region between the acceptor stem and D-stem, a striking feature of tRNAs from the Δ*pylSn* class of enzymes (37). Installing a U at this position (found in Mm tRNA^Pyl^, variant Ma07) was also found to decrease activity *in vitro* (*P* < 0.01, Figure 2B) and *in vivo* (*P* < 0.0001, Figure 2C). Furthermore, while the A-bulge did not have an overall effect on function of Ma tRNA^Pyl^, a G:U wobble pair (G28:U42) is found just below that in the anticodon stem. This differs from Mm tRNA^Pyl^ which has a U:G wobble pair in this same position. Swapping the position of the G and U (variant Ma08), an action known to affect structure (38), negatively impacted the recognition capability of MaPylRS to the tRNA (Figure 2). These data highlights four regions, focused near or in the acceptor stem and in the anticodon stem, which are responsible for promoting recognition of MaPylRS.

### Installing tRNA^Pyl^ elements promotes activity with MaPylRS

**Figure 3.**
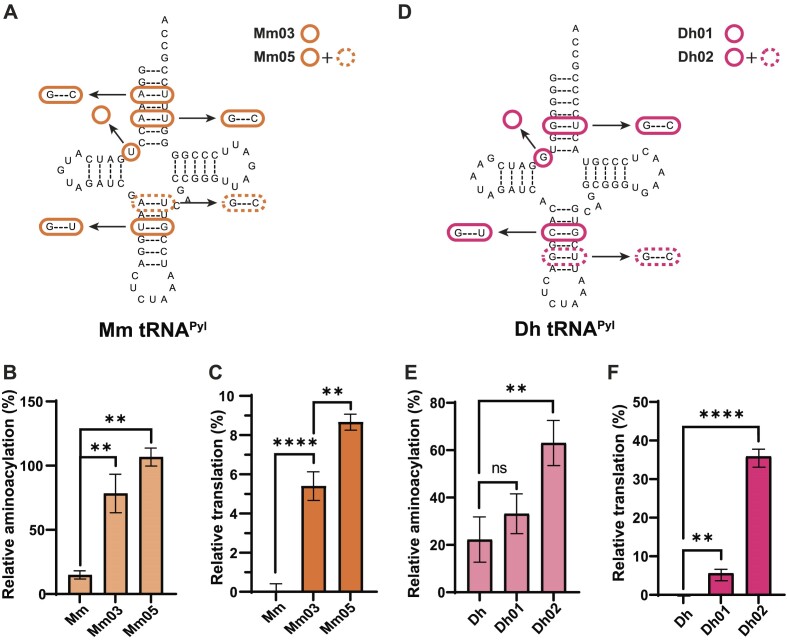
Transfer of identity elements into other tRNA^Pyl^ sequences promotes MaPylRS recognition. (**A**) Mm tRNA^Pyl^ variant cloverleaf structures. Bases that are changed or missing are represented with an orange circle. (**B**) *In vitro* assays with Mm tRNA^Pyl^ variants show the effect of tRNA mutations on aminoacylation efficiency with MaPylRS. (**C**) *In vivo* fluorescent readthrough assays with Mm tRNA^Pyl^ variants show the effect of tRNA mutations on translation with MaPylRS. (**D**) Dh tRNA^Pyl^ variant cloverleaf structures. Bases that are changed or missing are represented with a pink circle. (**E**) *In vitro* assays with Dh tRNA^Pyl^ variants show the effect of tRNA mutations on aminoacylation efficiency with MaPylRS. (**F**) *In vivo* fluorescent readthrough assays with Dh tRNA^Pyl^ variants show the effect of tRNA mutations on translation with MaPylRS. Data shown is the average of at least three biological replicates with the error bars representing the standard deviation. Percent activity is calculated with Ma tRNA^Pyl^ at 100%. Statistical analysis performed with a paired t-test and significance represented as stars.

To confirm that we identified all the Ma tRNA^Pyl^ elements necessary for recognition by MaPylRS, we transferred them into a tRNA^Pyl^ that MaPylRS does not recognize. We started with Mm tRNA^Pyl^ as a scaffold and inserted G3:C70, G5:C68, and G28:U42 in place of the bases that were originally there (A3:U70, A5:U68, and U28:G42) and subsequently removed U8 (Figure 3A, variant Mm03). Through engineering in these changes, we were able to induce recognition by MaPylRS. The *in vitro* aminoacylation activity of Mm03 increased roughly 5-fold (from 15% to 78% relative to Ma, *P* < 0.01, Figure 3B) while *in vivo* readthrough of sfGFP reached 5% from 0% (*P* < 0.0001, Figure 3C). In attempts to further improve *in vivo* activity, an additional change of A26:U44 to G26:C44 was made (variant Mm05). This further increased both the *in vitro* and *in vivo* activity to 106% (*P* < 0.01) and 9% (*P* < 0.01), respectively. The inherently low activity of Mm tRNA^Pyl^ in *E. coli*, even with its cognate synthetase (MmPylRS) (Figure 1B, C), may be the reason that we were unable to increase the translation efficiency with MaPylRS any further.

The tRNA^Pyl^ from the *pylSn* class are also not recognized by MaPylRS, therefore we tested if our identified Ma tRNA^Pyl^ elements could promote interaction with MaPylRS. We chose to work with tRNA^Pyl^ from *D. hafniense* due to previous characterization of its interaction with its cognate PylRS (7,39,40). Furthermore, the similarity of Dh tRNA^Pyl^ compared to Ma tRNA^Pyl^ required only three changes (G5:C68, G28:U42 and removal of G8) to achieve the same transferred tRNA elements as in Mm03 (Figure 3D, variant Dh01). However, the effect of these changes on activity with MaPylRS were not convincing with *in vitro* aminoacylation efficiency being not statistically significantly different from the original Dh tRNA^Pyl^ (Figure 3E) and the *in vivo* sfGFP readthrough translation efficiency only increased to 6% (*P* < 0.01, Figure 3F). Therefore, we took a closer look at the sequence of all three tRNAs to determine what differed between Ma and Mm tRNA^Pyl^. From this we found an additional G:U wobble pair (G30:U40) in the anticodon stem of Dh tRNA^Pyl^. This combined with the addition of G28:U42 could affect the stacking of the anticodon stem, interfering with efficient recognition by MaPylRS (41). Upon mutating G40 to C40, to form a Watson-Crick base pair (variant Dh02), the aminoacylation (63%, *P* < 0.01) and translation (36%, *P* < 0.0001) efficiency were increased (Figure 3E, F). To confirm whether the A-bulge, that was originally removed from the Ma tRNA^Pyl^ sequence would increase activity of these new Mm and Dh tRNAs, found mixed results. Adding in A42a to Mm05 (Mm05A) showed a 10% increase in *in vivo* GFP production (*P* < 0.0001), while in Dh02A, the activity was decreased by 25% (*P* < 0.0001) (Supplementary Figure S1).

### Cryo-EM imaging reveals impact of Ma tRNA^Pyl^ identity elements on tRNA shape

**Figure 4.**
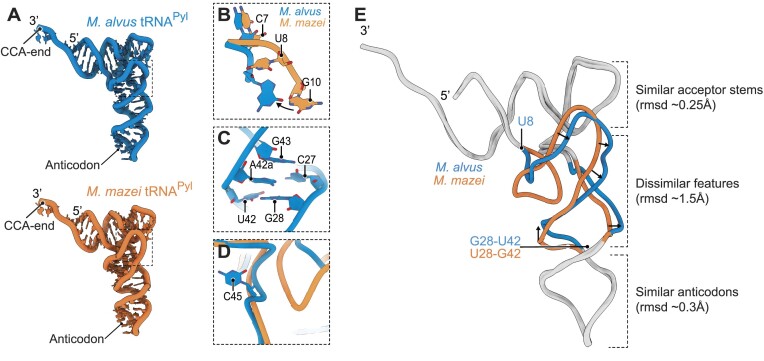
Cryo-EM imaging reveals an impact of Ma tRNA^Pyl^ identity elements on tRNA shape. (**A**) Overall structures of tRNA^Pyl^ from *M. alvus* (blue) and *M. mazei* (orange). (**B**) A view of superposed structures tRNA^Pyl^ from *M. alvus* and *M. mazei* illustrates the absence of a nucleotide at position 8 in tRNA^Pyl^, leading to a change in positioning of conserved bases in this tRNA segment. (**C**) A view of the anticodon stem of Ma tRNA^Pyl^ illustrates that, unlike previous predictions, base A42a does not bulge out of the tRNA body and instead intercalates into the anticodon stem. (**D**) A view of superposed structures highlights the C45 bulge in Ma tRNA^Pyl^. (**E**) A complete overlay of Ma tRNA^Pyl^ and Mm tRNA^Pyl^ shows the dissimilar features that arise between the identity elements U8 and G28:U42.

To reveal the mechanism by which the Ma tRNA^Pyl^ identity elements mediate highly specific recognition of Ma tRNA^Pyl^ by MaPylRS, we determined two cryo-EM structures. We achieved high resolution (2.8–2.9Å) maps by using the ribosome as a scaffold for visualizing Ma or Mm tRNA^Pyl^ (Figure 4A, Supplementary Figure S2), a strategy that we previously showed to be successful (13). Comparison of these two structures revealed four unique structural features of Ma tRNA^Pyl^ compared to Mm tRNA^Pyl^, providing insight into its orthogonal recognition by MaPylRS.

The first major difference is the absence of a base at position 8 (at the acceptor/D-stem junction). This missing base causes changes in the overall structure of the linker between the acceptor stem and the D-stem. In Mm tRNA^Pyl^, as in other tRNAs, the presence of U8 pushes G10 into a loop. However in its absence (Ma tRNA^Pyl^), G10 resides right next to C7, resulting in an atypical conformation of bases C7 and G10 (Figure 4B).

A second difference is the A42a-bulge, named for its appearance as a bulge in the Ma tRNA^Pyl^ secondary structure due to its inability to base-pair with either C27 or G28 in the anticodon stem. Our tertiary structure reveals that this bulge is not visually present. Instead, A42a is sandwiched between two base pairs, C27:G43 and G28:U42. As an apparent consequence of this intercalation of A42a into the acceptor stem of the tRNA molecule, we observed an upward shift of nucleotides 43 and 44 towards the acceptor stem, altering the tRNA structure (Figure 4C).

Thirdly, Ma tRNA^Pyl^ has a pronounced C45 that is missing in the ribosome-bound Mm tRNA^Pyl^. A similar bulge has been previously seen in the crystal structure of Mm tRNA^Pyl^ (PDBID: 5UD5) (9) (Supplementary Figure S3, Figure 4D). The reason for the difference in this region for Mm tRNA^Pyl^ is unclear, however we do know that position 45 is the first nucleotide in the variable arm. Previous studies have shown the importance of variable arm structure in the ribosome for efficient translation, and we know that in *E. coli* Mm tRNA^Pyl^ does not translate as well as Ma tRNA^Pyl^ (13,42,43) (Figures 1 and 3C).

Finally, by overlaying the two tRNA^Pyl^ structures obtained from cryo-EM imaging on the ribosome, we found a nearly identical conformation of their acceptor stems and lower part of their anticodon stems (rmsd of 0.25–0.30 Å between their backbone atoms). However, the two tRNA structures began to deviate from each other (rmsd ∼1.5 Å between their backbone atoms) within a stretch between U8 and U28 in Mm tRNA^Pyl^ (Figure 4E). This overall shape change appears to be caused by the elements in Ma tRNA^Pyl^ which we determined to be necessary for MaPylRS recognition; the deletion of U8 in Ma tRNA^Pyl^ truncating the loop between the acceptor stem and D-stem, and the mutation of U28:G42 to G28:U42 (and possibly the A42a intercalation) affecting the trajectory of the phosphate backbone (38). Taken together, our structural analysis suggests that these two identified Ma tRNA^Pyl^ elements act by changing the overall shape of the tRNA molecule.

### Simulations reveal MaPylRS recognition requires rigid identity structure

**Figure 5.**
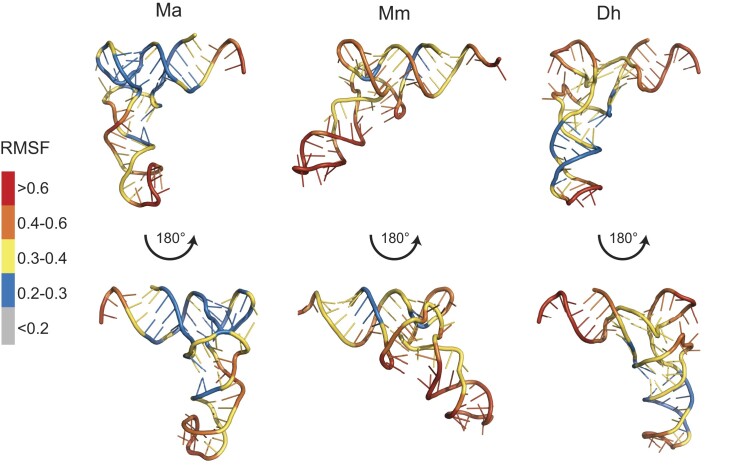
Molecular dynamic simulations reveal rigidity of Ma tRNA^Pyl^. Shown is the averaged simulated structure of each tRNA^Pyl^, colored according to the residues’ root mean squared fluctuations (RMSF). Red is high fluctuation (>0.6) and grey is low fluctuation (<0.2).

The activity assays and structural data both emphasize the importance of G28:U42 and the absence of a base at position 8 for MaPylRS recognition. The structural data, however, does not illuminate how the G:C base pairs in the acceptor stem and anticodon stem facilitate this interaction. Furthermore, the Dh tRNA^Pyl^ variant data showed that simply transplanting G28:U42 and removing a base in position 8 is not sufficient to promote activity with MaPylRS (Figure 3D–F, Supplementary Figure S4A). To unravel this, we performed molecular dynamic simulations of the tRNA^Pyl^ variants in solution. We found that Ma tRNA^Pyl^ adopts the canonical L-shaped tertiary structure with low RMSF values at the L-junction and increased RMSF at the 3′-CCA end and anticodon loop (Figure 5). On the other hand, Mm tRNA^Pyl^ adopts an obtuse angle with increased RMSF throughout the tRNA including the L-junction. For Dh tRNA^Pyl^ we used the tRNA crystal structure (PDBID:2ZNI) as a starting point for the simulation and observed that the overall shape resembles the canonical L-shape that is expected, with low RMSF in the anticodon region only and mid RMSF values in the L-junction (Figure 5). These simulations suggest that MaPylRS requires a rigid L-shaped tRNA structure for efficient recognition.

To test this hypothesis of a requirement of tRNA rigidity, we also simulated the tRNA^Pyl^ variants we studied. Without a high-resolution structure for each variant, we generated starting structures through the mutagenesis tool in PyMOL. Starting with removal of the A-bulge for Ma02, we found a slight fluctuation increase in the acceptor domain and a rearrangement in stability for the anticodon stem. In Ma tRNA^Pyl^, there is high flexibility at the A-bulge while the opposite side of the anticodon stem is more rigid. For Ma02, the fluctuation is now moderate (0.3–0.4) on both sides of the A-bulge (Supplementary Figure S4). With this for comparison, simulations were also performed for Ma tRNA^Pyl^ variants that were poorly recognized by MaPylRS. In all variants, the flexibility of the tRNA was found to increase, mainly in the anticodon stem and in some cases also the acceptor stem, with minimal changes to the L-junction stability (Supplementary Figure S4B). This further emphasizes the requirement of a rigid tRNA^Pyl^ for MaPylRS activity.

**Figure 6.**
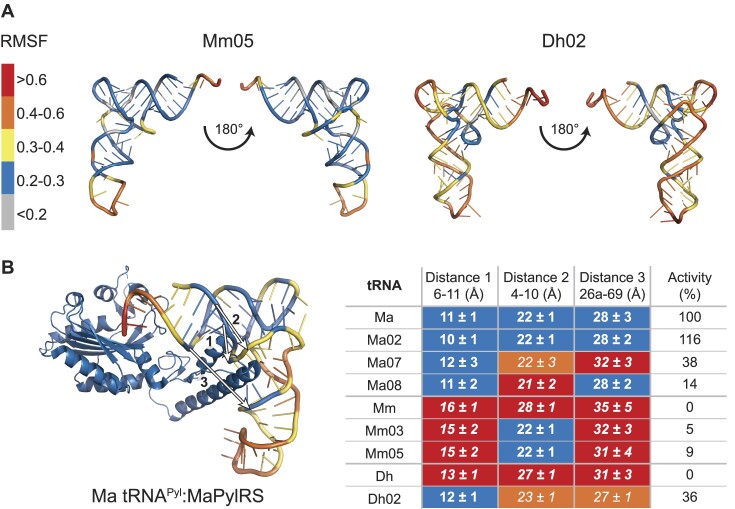
Mutations to tRNA^Pyl^ induce rigidity, conforming these tRNAs to an ideal structure for recognition by MaPylRS. (**A**) tRNA^Pyl^ variants Mm05 and Dh02 were found to be more rigid and have a canonical L-shaped compared to their parent tRNA^Pyl^ (Mm and Dh, respectively). (**B**) Measuring three distances at the proposed Ma tRNA^Pyl^:MaPylRS interface (PDBID:6EZD) revealed the necessary distances required for maximal activity with MaPylRS. Distance 1 is shown to be the major contributor in this recognition.

As we saw with the activity assays, the major question was whether the mutations identified to induce recognition of Mm and Dh tRNA^Pyl^ by MaPylRS did so by inducing rigidity to the tRNA. Strikingly, simulations of Mm05 showed a more canonical L-shaped structure compared to Mm, with increased rigidity (0.2–0.3) (Figure 6A). A similar observance was seen with Dh02, in which the L-shape structure shifted to ∼90° compared to ∼120° (Figure 6A). This confirmed our hypothesis that the general requirement for MaPylRS recognition is a rigid L-shaped tRNA.

The simulations provide a clear visual explanation for why MaPylRS recognizes Ma, Ma02, Mm05 and Dh05, but not any of the other tRNA^Pyl^ variants. However, it does not clarify why the *in vivo* activity of Mm05 is poor compared to Dh02. To further investigate this, we considered the tRNA interaction site of PylRS. Based on the complex structure of Dh tRNA^Pyl^ with the C-terminal domain of its cognate PylRS we chose six nucleotides in the tRNA binding pocket (36,40). We noted these nucleotides on our tRNA^Pyl^ variants and measured the distances (nucleotides 6–11, 4–10 and 26a-69) between them using the phosphorus atom from the phosphate backbone of the averaged simulated tRNA structures (Figure 6B). From this, we found that as these three distances approach (within 1 Å) those for Ma tRNA^Pyl^, the translation activity with MaPylRS increases (Figure 6B). Moreover, the distance between nucleotide 6 and 11 appears critical for *in vivo* activity. A 1 Å decrease in distance for Ma02 compared to Ma, improved activity to 116% that of Ma tRNA^Pyl^. On the other hand, even though Mm05 has equivalent distances for Ma tRNA^Pyl^ between nucleotides 4–10 and 26a-69, the 4 Å wider distance between nucleotides 6–11 limited *in vivo* activity to only 9% that of Ma tRNA^Pyl^. A multiple linear regression analysis revealed correlation coefficients of –0.68, –0.43 and –0.58 for distance 1, 2 and 3, respectively. This emphasizes the stronger impact of distance 1 on the activity with MaPylRS. Given that we measured averaged structures; we also plotted the distribution of distances that are present throughout the simulation (Supplementary Figure S5-7). For tRNA^Pyl^ variants that are recognized well by MaPylRS, there was a tendency for a narrow distribution of distances centred at the correct distance (11 Å for distance 1, 22 Å for distance 2 and 28 Å for distance 3) while variants that were poorly recognized either had a wider distribution of distances, or a narrow distribution centred at a larger distance (Supplementary Figure S5-7).

### MaPylRS is monomeric at physiological concentrations

**Figure 7.**
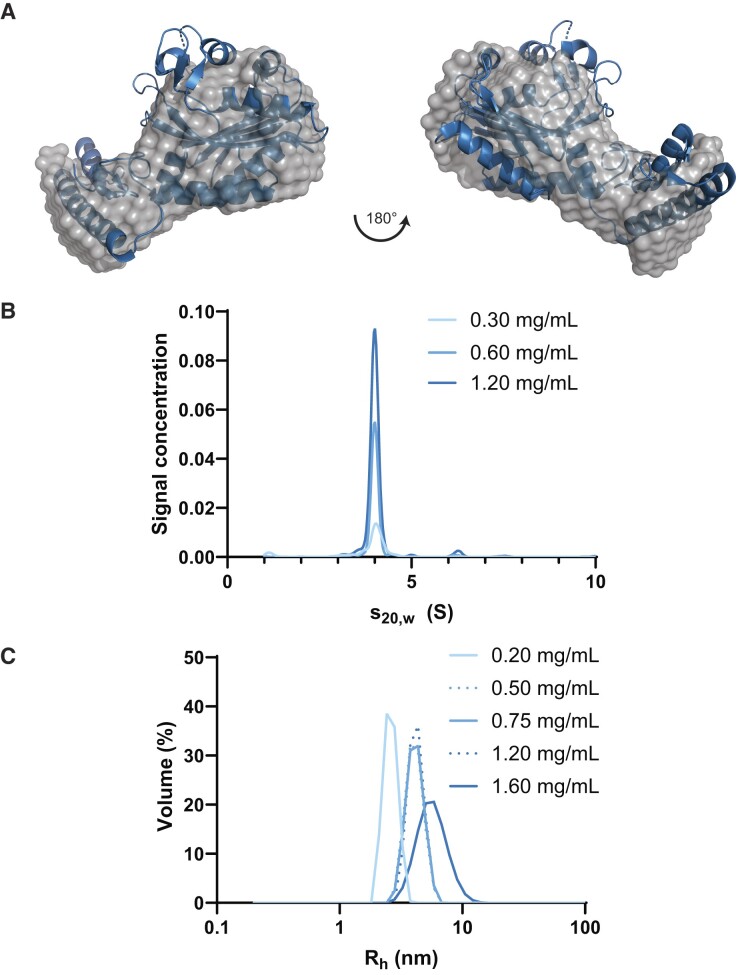
Biophysical characterization reveals dynamic behavior of MaPylRS. (**A**) *Ab initio* models generated from HPLC-SAXS of MaPylRS overlaid with the crystal structure (PDBID:6EZD) shows the monomeric nature of this enzyme. (**B**) AUC data at multiple concentrations revealed a strong dimeric presence of the enzyme. (**C**) Light scattering at a wider range of concentrations revealed three distinct hydrodynamic radii (*R*_h_).

To further explain the requirement for a structurally rigid tRNA^Pyl^ for interaction, we biophysically characterized MaPylRS in solution. SAXS data showed that MaPylRS exists as a monomer in solution (Figure 7A) with a 21 Å radius of gyration (*R*_g_) and 62 Å maximum particle diameter (*D*_max_) (Table 1). Given that the crystal structures (PDBID: 6EZD and 6JP2) suggest MaPylRS as a dimer, we investigated the concentration dependence of the oligomerization state. AUC (Figure 7B) and DLS (Figure 7C) data confirmed that at low μM concentrations (6 μM [0.2 mg/ml]), MaPylRS is found as a monomer. Plotting the *R*_h_ at a range of concentrations showed clear oligomerization to a dimer state by 16 μM (0.5 mg/ml), based on hydrodynamic predictions from the crystal structure using HullRad (Table 1) (44), and further oligomerization by 50 μM (1.6 mg/ml) (Figure 7C).

**Table 1.** Hydrodynamic parameters of MaPylRS from experimental and theoretical conditions

Parameters	MaPylRS	Monomer^g^	Dimer^g^
*M_W_* (kDa)^a^	32.15	30.49	61.15
Guinier *R_g_* (Å)	21.59 ± 0.06	21.47	25.35
*P*(*r*) *R*_g_ (Å)^b^	21.00 ± 0.02	–	–
*D*_max_ (Å)^b^	62	74.87	100.80
$\chi^2$ ^c^	1.8	–	–
NSD^d^	0.76	–	–
*R*_h_ (Å)^e^	26 ± 2	26.83	34.07
*s°*_20,w_ (S)^e^	2.45	2.65	4.18
*M*_w_ (kDa)^f^	32	30.49	61.15

^a^Calculated from amino acid/nucleotide sequence.

^b^Experimentally measured using GNOM analysis from SAXS data.

^c^Goodness-of-fit parameter between raw data and data back-calculated from *ab initio* model.

^d^Normalized spatial discrepancy indicating agreement between models.

^e^Experimentally measured from AUC.

^f^Calculated from *R_h_* and *s°*_20,w_.

^g^Determined from HullRad based on PDBID:6EZD.

## Discussion

In this study, we unveiled the key nucleotides in tRNA^Pyl^ that are required for recognition of *M. alvus* PylRS. Our initial goal to find the tRNA identity elements which interacted with PylRS from the Δ*pylSn* class, revealed that these tRNA elements together formed a distinct structure which is recognized (45). This identity structure is found to be induced by the absence of a base at position 8 and the presence of a wobble base pair, G28:U42. However, these two elements are not sufficient for recognition by MaPylRS and must be complimented by additional G:C pairs (up to three) in both the acceptor and anticodon stem. We showed that combining these tRNA elements into other tRNA^Pyl^ which are not recognized by MaPylRS (from the *pylSn-pylSc* and *pylSn* class) promoted aminoacylation by MaPylRS and subsequent protein translation.

Through MD simulation we explain that these five elements induce rigidity (lowers RMSF) and promotes formation of a distinct tRNA identity structure recognized by MaPylRS. The rigid identity structure of Ma tRNA^Pyl^, suggests coevolution of Δ*pylSn* class PylRS enzymes and their cognate tRNAs (4). Starting with the *pylSn-pylSc* class of enzymes where the NTD is physically connected to the CTD with a linker, the cognate tRNA (Mm tRNA^Pyl^) showed a high RMSF with an averaged structure that appeared like more of an obtuse angle than an L-shape. As these two genes separate (*pylSn* class), we observed that the cognate tRNA (Dh tRNA^Pyl^) became more rigid and L-shaped, but not as rigid as tRNA^Pyl^ from the Δ*pylSn* class where there is no NTD. The NTD of PylRS has previously been identified as a recruitment domain, having high affinity for tRNA^Pyl^ (7). Our data suggests that in addition to recruiting tRNA^Pyl^, the NTD may act to stabilize it in solution to promote binding of the CTD and subsequent aminoacylation. Without an NTD, it follows that MaPylRS requires a tRNA that is already stabilized in the correct orientation, ready to be recognized and aminoacylated.

This hypothesis is amplified upon characterizing the oligomerization of MaPylRS. At physiological concentrations, MaPylRS is monomeric. This is in contrast to what is suggested for DhPylRS (36) and characteristic of most other class II aaRSs (5). Given that each copy of DhPylRS interacts with both Dh tRNA^Pyl^ molecules, a monomeric enzyme would be missing this additional stability for complex formation (36). Moreover, we have been unable to isolate a Ma tRNA^Pyl^:MaPylRS complex in solution, potentially owing to Ma tRNA^Pyl^ being structurally ready for aminoacylation.

PylRS translation systems have been heavily studied for their use in genetic code expansion with emphasis on developing multiple orthogonal tRNA^Pyl^:PylRS pairs. The recent identification of three classes of ΔN tRNA^Pyl^ has expanded the use of PylRS to five orthogonal systems (37,46). Our identification of the elements required for MaPylRS recognition provides understanding for this orthogonality and also suggests an evolutionary relationship between Ma tRNA^Pyl^ and MaPylRS. Moreover, the notion that mutations in tRNA can affect its flexibility in solution may provide insights into development of tRNA therapeutics.

## Supplementary Material

gkad1188_supplemental_file

## Data Availability

Atomic coordinates and structure factors for the reported cryo-EM structures have been deposited with the Protein Data Bank under accession numbers 8UPT and 8UPY and the Electron Microscopy Data Bank under accession numbers EMD-42455 and EMD-42457.

## Notes

Present address: Jeffery M. Tharp, Department of Biochemistry and Molecular Biology, Indiana University School of Medicine, Indianapolis, IN 46202, USA.

Present address: Natalie Krahn, Department of Biochemistry and Molecular Biology, University of Georgia, Athens, GA 30602, USA.
