# Supplementary material for: tRNA shape is an identity element for an archaeal pyrrolysyl-tRNA synthetase from the human gut
Source: Nucleic Acids Res. 2023 Dec 15;52(2):513–24. doi: 10.1093/nar/gkad1188 (PMC10810272; doi:10.1093/nar/gkad1188)
Supplement: gkad1188_supplemental_file [file gkad1188_supplemental_file.pdf]

SUPPLEMENTARY DATA

tRNA shape is an identity element for an archaeal pyrrolysyl-tRNA synthetase from the human gut

Natalie Krahn[1,†], Jingji Zhang[2,†], Sergey V. Melnikov[3], Jeffery M. Tharp[1], Alessandra Villa[4], Armaan Patel[1], Rebecca J Howard[5], Haben Gabir[6], Trushar R. Patel[7,8,9], Jörg Stetefeld[6,10], Joseph Puglisi[2] and Dieter Söll[1,11*]

[1] Department of Molecular Biophysics and Biochemistry, Yale University, New Haven, CT 06520, USA
[2] Department of Structural Biology, Stanford University School of Medicine, Stanford, CA 94305, USA
[3] Biosciences Institute, Newcastle University, Newcastle upon Tyne, UK
[4] PDC-Center for High Performance Computing, KTH-Royal Institute of Technology, Stockholm, Sweden
[5] Department of Biochemistry and Biophysics, Science for Life Laboratory, Stockholm University, Solna, Sweden
[6] Department of Chemistry, University of Manitoba, Winnipeg, MB R3T 2N2, Canada
[7] Department of Chemistry and Biochemistry, Alberta RNA Research and Training Institute, University of Lethbridge, Lethbridge, AB T1K 2E1, Canada
[8] Li Ka Shing Institute of Virology, University of Alberta, Edmonton, AB T6G 2E1, Canada
[9] Department of Microbiology, Immunology & Infectious Diseases, Cumming School of Medicine, University of Calgary, Calgary, AB T2N 4N1, Canada
[10] Department of Microbiology, University of Manitoba, Winnipeg, MB R3T 2N2, Canada
[11] Department of Chemistry, Yale University, New Haven, CT 06520, USA

* To whom correspondence should be addressed. Tel: 1 (203) 432-6200; Email: dieter.soll@yale.edu

[†]These authors contributed equally to the work

Present Addresses:
Jeffery M. Tharp, Department of Biochemistry and Molecular Biology, Indiana University School of Medicine, Indianapolis, IN 46202, USA

Natalie Krahn, Department of Biochemistry and Molecular Biology, University of Georgia, Athens, GA 30602, USA

This PDF includes:

Supplementary Figures S1-S7

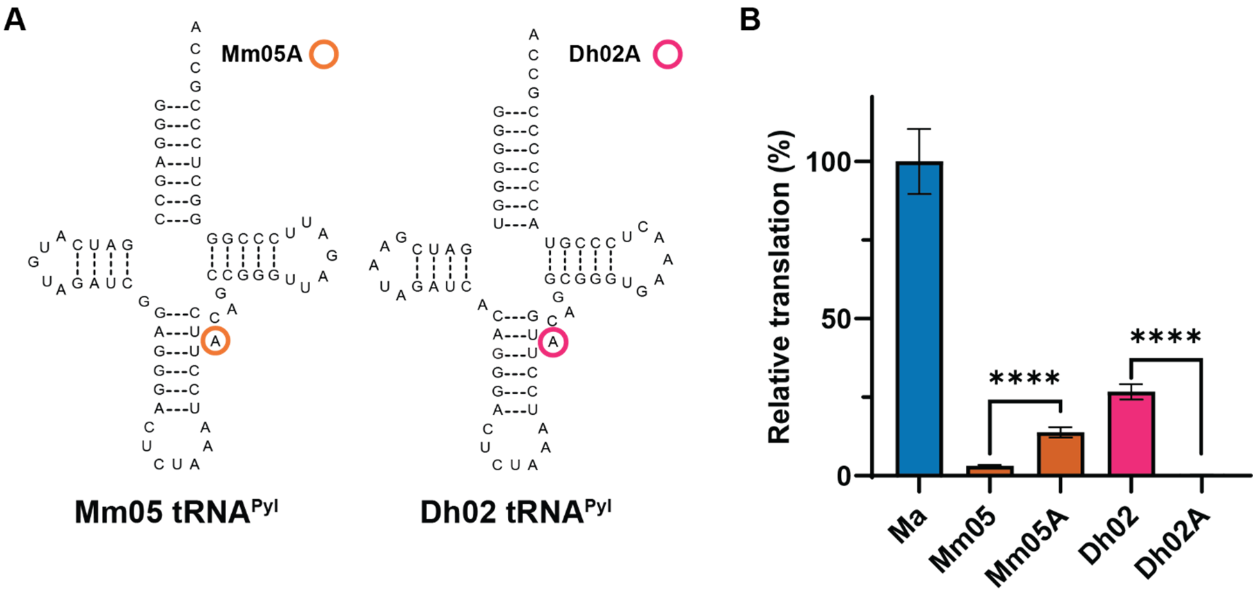

Supplementary Figure S1. Transfer of A42a bulge into Mm05 and Dh02 $tRNA^{Pyl}$ sequences. (A) $tRNA^{Pyl}$ variant cloverleaf structures. (B) *In vivo* fluorescent readthrough assays show the effect of the A-bulge on translation with MaPylRS. Data shown is the average of at least three biological replicates with the error bars representing the standard deviation. Percent activity is calculated with Ma tRNAPyl at 100%. Statistical analysis performed with a paired t-test and significance shown as stars.

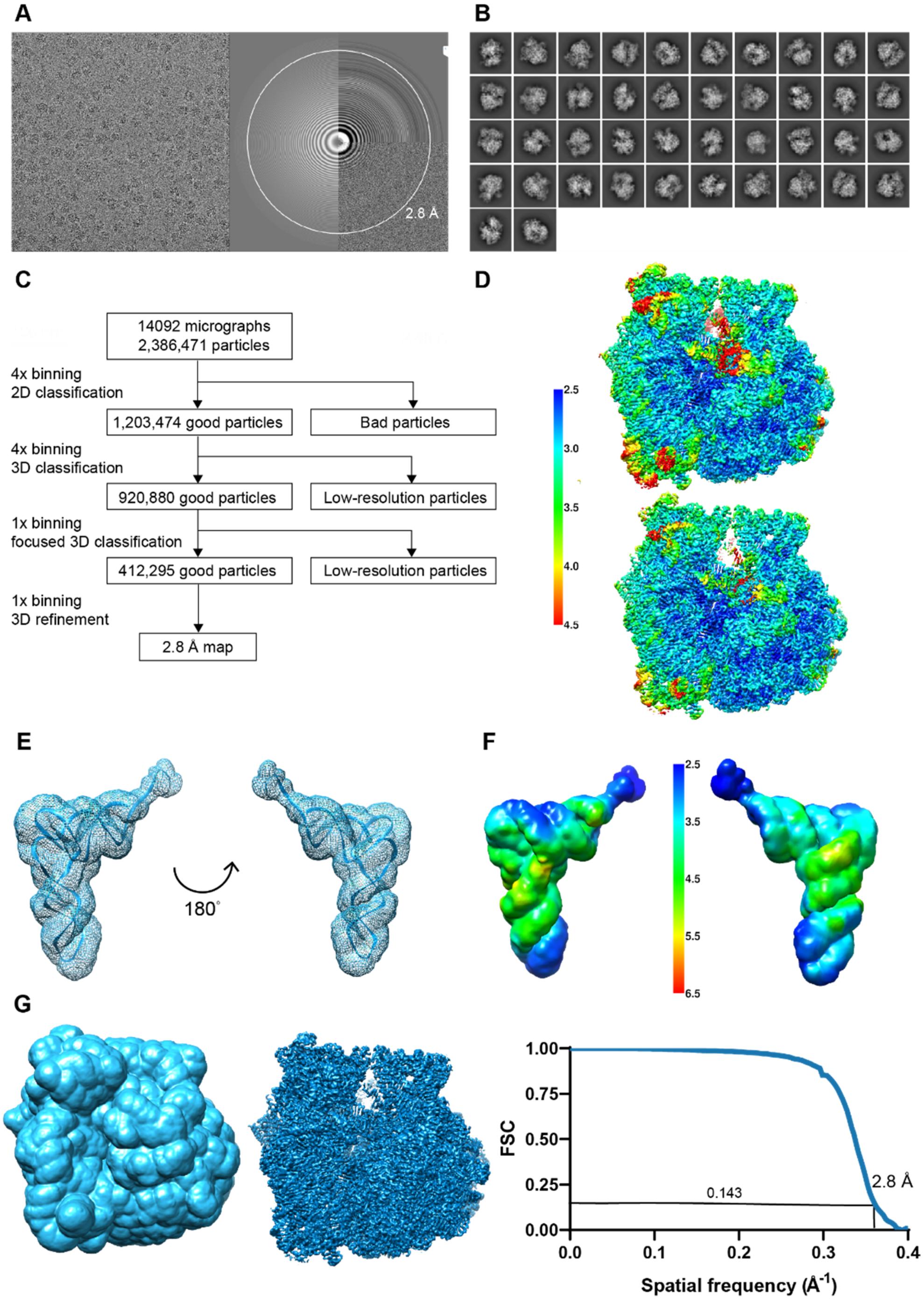

Supplementary Figure S2. Cryo-EM data processing of Ma tRNA^{Pyl} in complex with the ribosome. (**A**) Representative micrograph collected with a Titan Krios transmission electron microscope and corresponding power spectrum. (**B**) Representative 2D class averages from reference-free 2D classification. (**C**) Particle classification and structural refinement procedures used. (**D**) Local resolution estimation of the cryo-EM density map of the ribosome. The density map is displayed in surface representation and colored according to the local resolution (see color bar). (**E**) mA tRNA^{Pyl} in the A site of the ribosome. (**F**) Local resolution estimation of the cryo-EM density map of mA tRNA^{Pyl} in the A site of the ribosome. The density map is displayed in surface representation and colored according to the local resolution (see color bar). (**G**) The mask used for resolution estimation (left) including all components of the reconstruction. FSC curve for cryo-EM reconstruction (right).

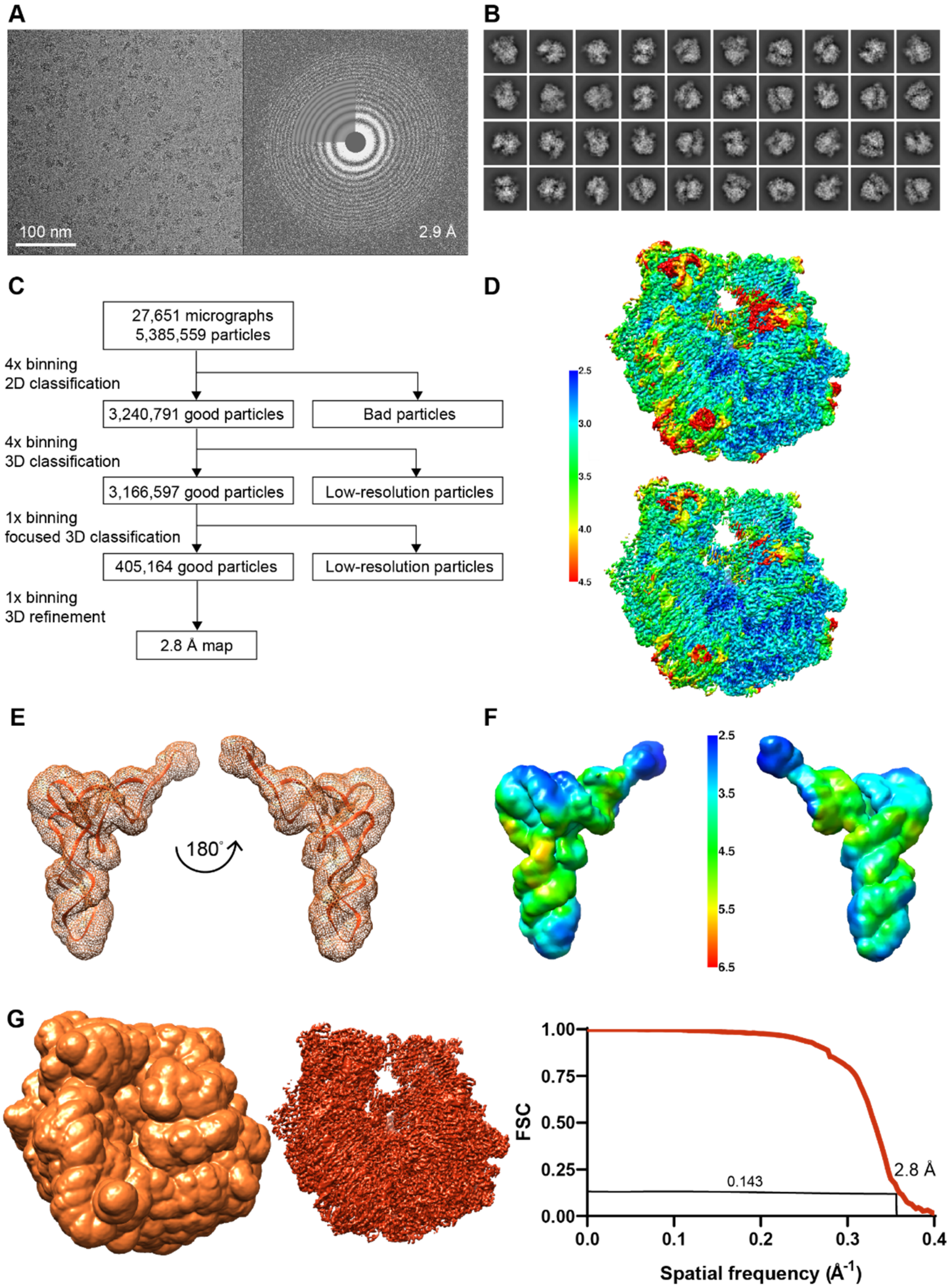

Supplementary Figure S3. Cryo-EM data processing of Mm tRNA^{Pyl} in complex with the ribosome. (**A**) Representative micrograph collected with a Titan Krios transmission electron microscope and corresponding power spectrum. (**B**) Representative 2D class averages from reference-free 2D classification. (**C**) Particle classification and structural refinement procedures used. (**D**) Local resolution estimation of the cryo-EM density map of the ribosome. The density map is displayed in surface representation and colored according to the local resolution (see color bar). (**E**) mM tRNA^{Pyl} in the A site of the ribosome. (**F**) Local resolution estimation of the cryo-EM density map of mM tRNA^{Pyl} in the A site of the ribosome. The density map is displayed in surface representation and colored according to the local resolution (see color bar). (**G**) The mask used for resolution estimation (left) including all components of the reconstruction. FSC curve for cryo-EM reconstruction (right).

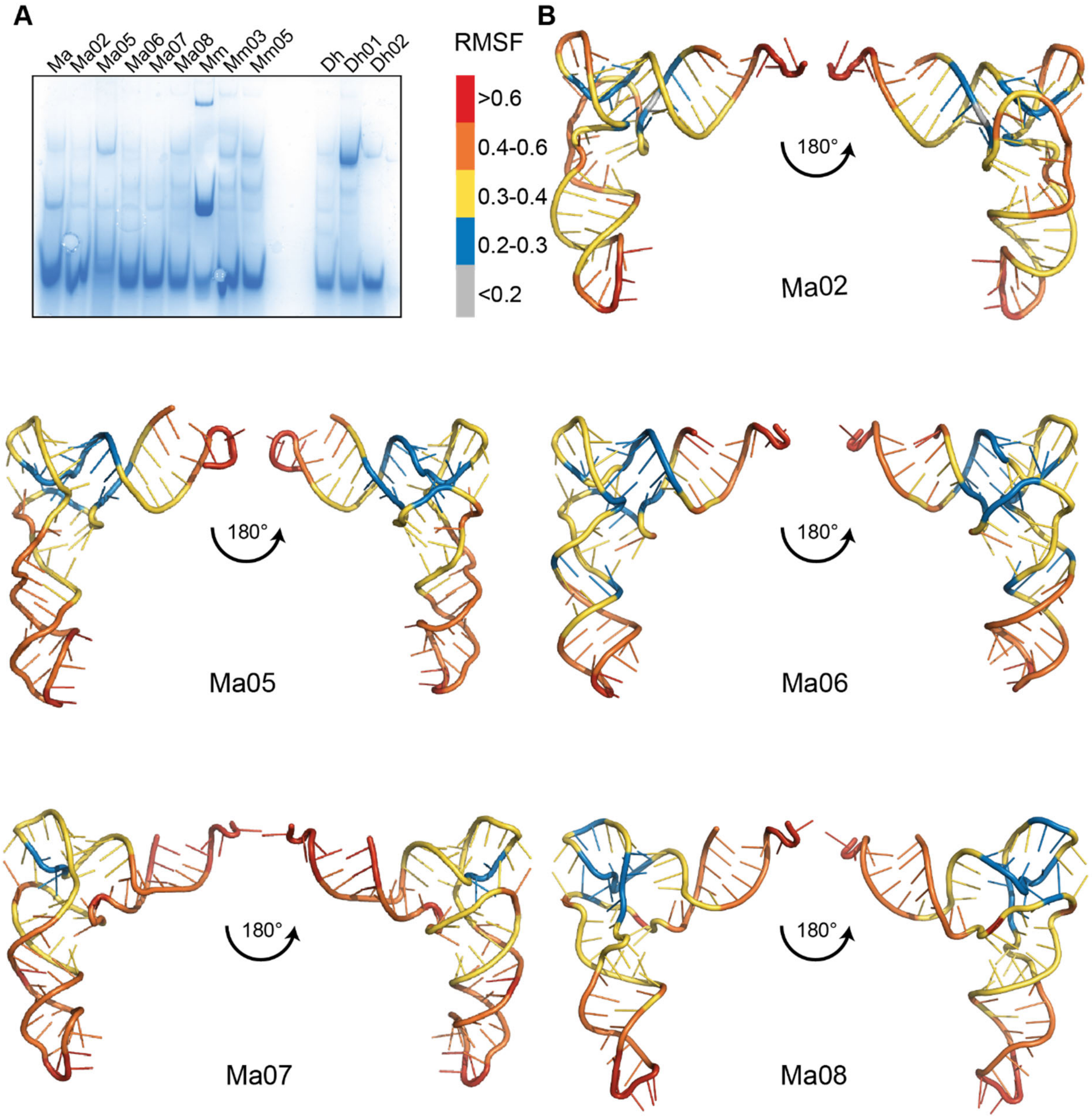

Supplementary Figure S4. Ma tRNA^{Pyl} variant characterization. (**A**) Native poly-acrylamide gel of all tRNA^{Pyl} variants. Mm and Dh01 have the largest population of higher molecular weight species. (**B**) Averaged simulations of the Ma tRNA^{Pyl} variants show decreased rigidity around the mutation area. Root mean squared fluctuation (RMSF) values were calculated for each nucleotide and colored accordingly (see color bar).

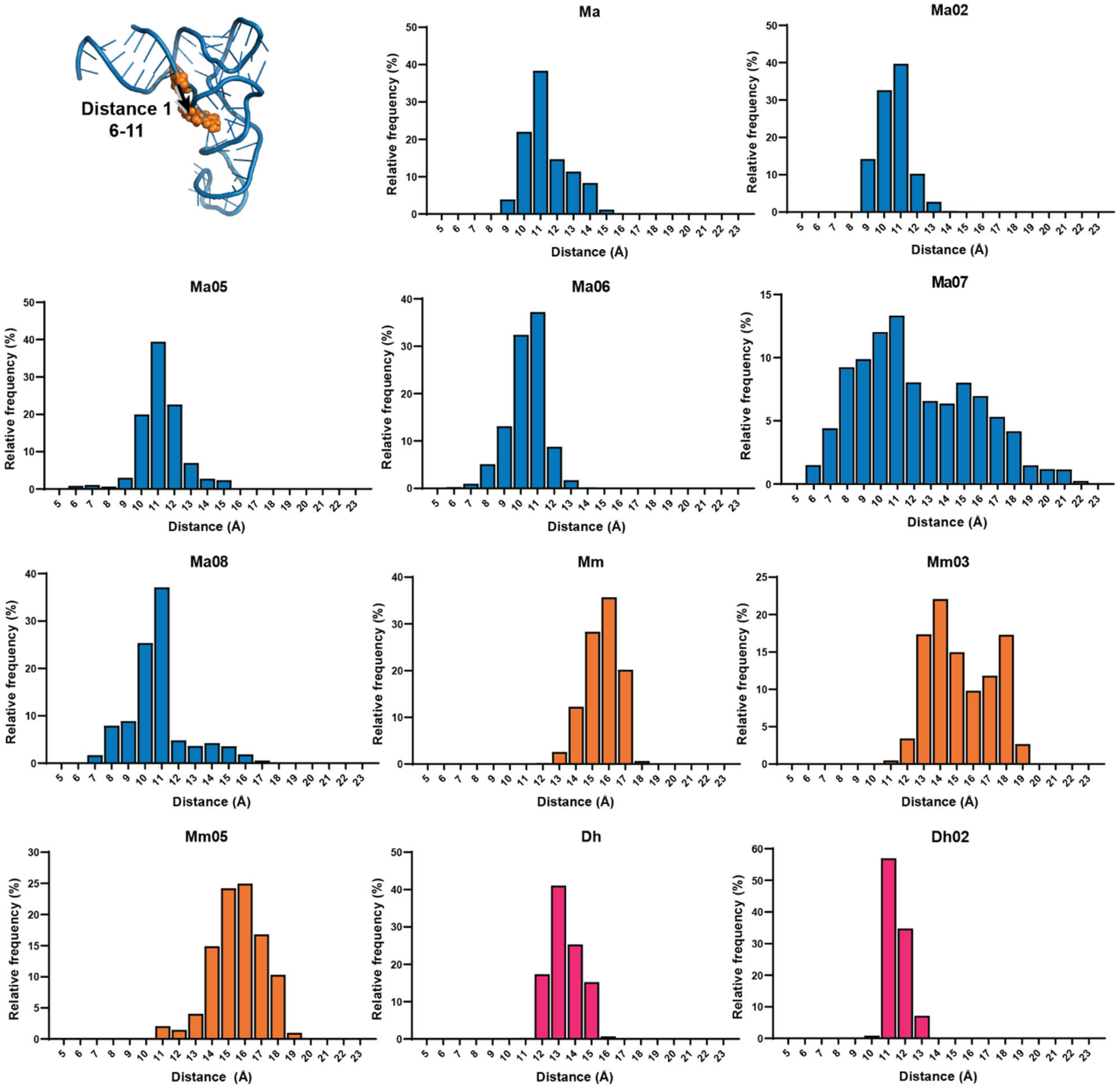

Supplementary Figure S5. Distance distribution of the simulated tRNA^{Pyl} variants from nucleotide 6-11 (distance 1). tRNA^{Pyl} variants that are well-recognized by MaPylRS have a narrow distribution centered around 10.5 Å.

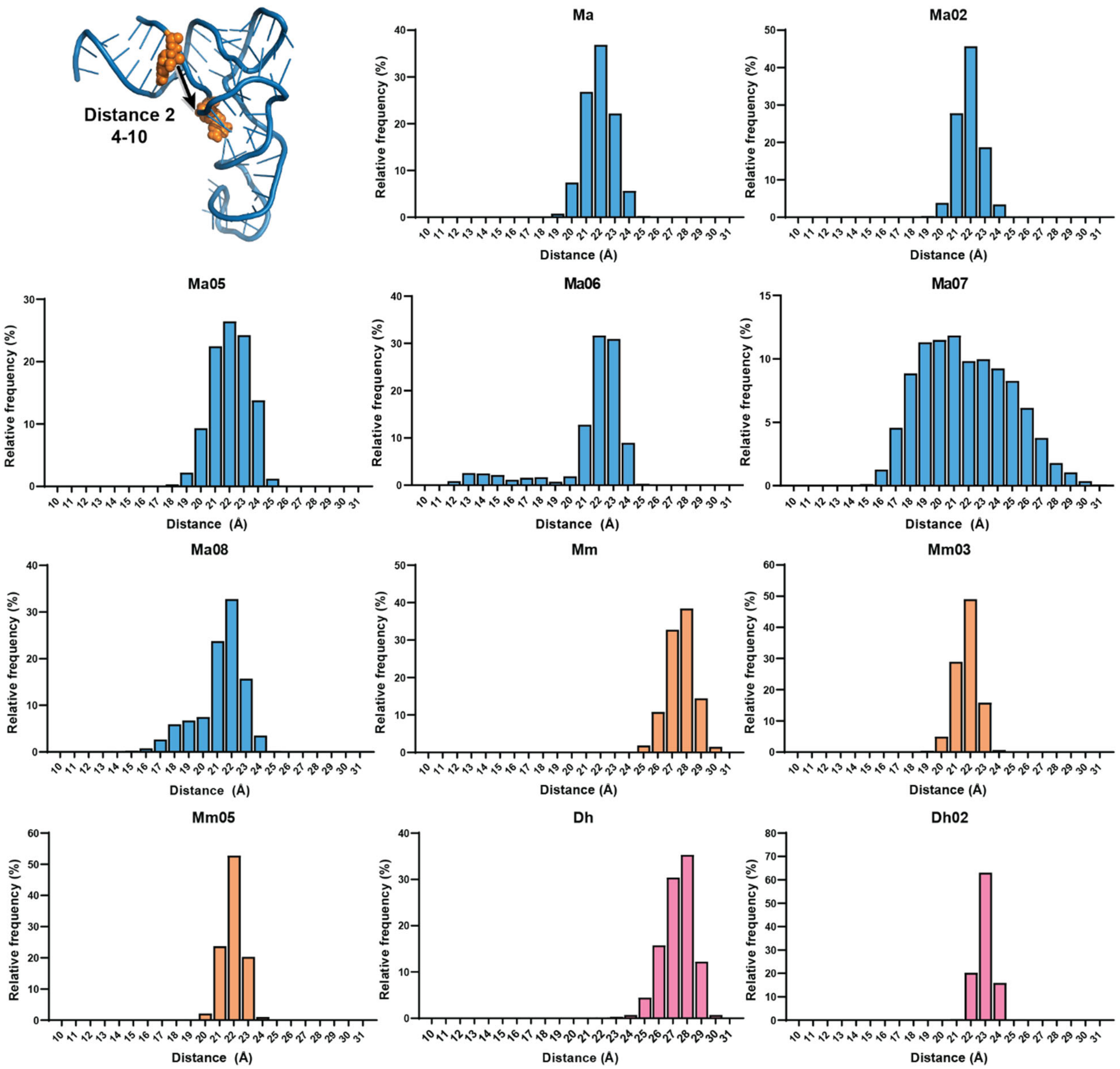

Supplementary Figure S6. Distance distribution of the simulated tRNA^{Pyl} variants from nucleotide 4-10 (distance 2). tRNA^{Pyl} variants that are well-recognized by MaPylRS have a narrow distribution centered around 22 Å.

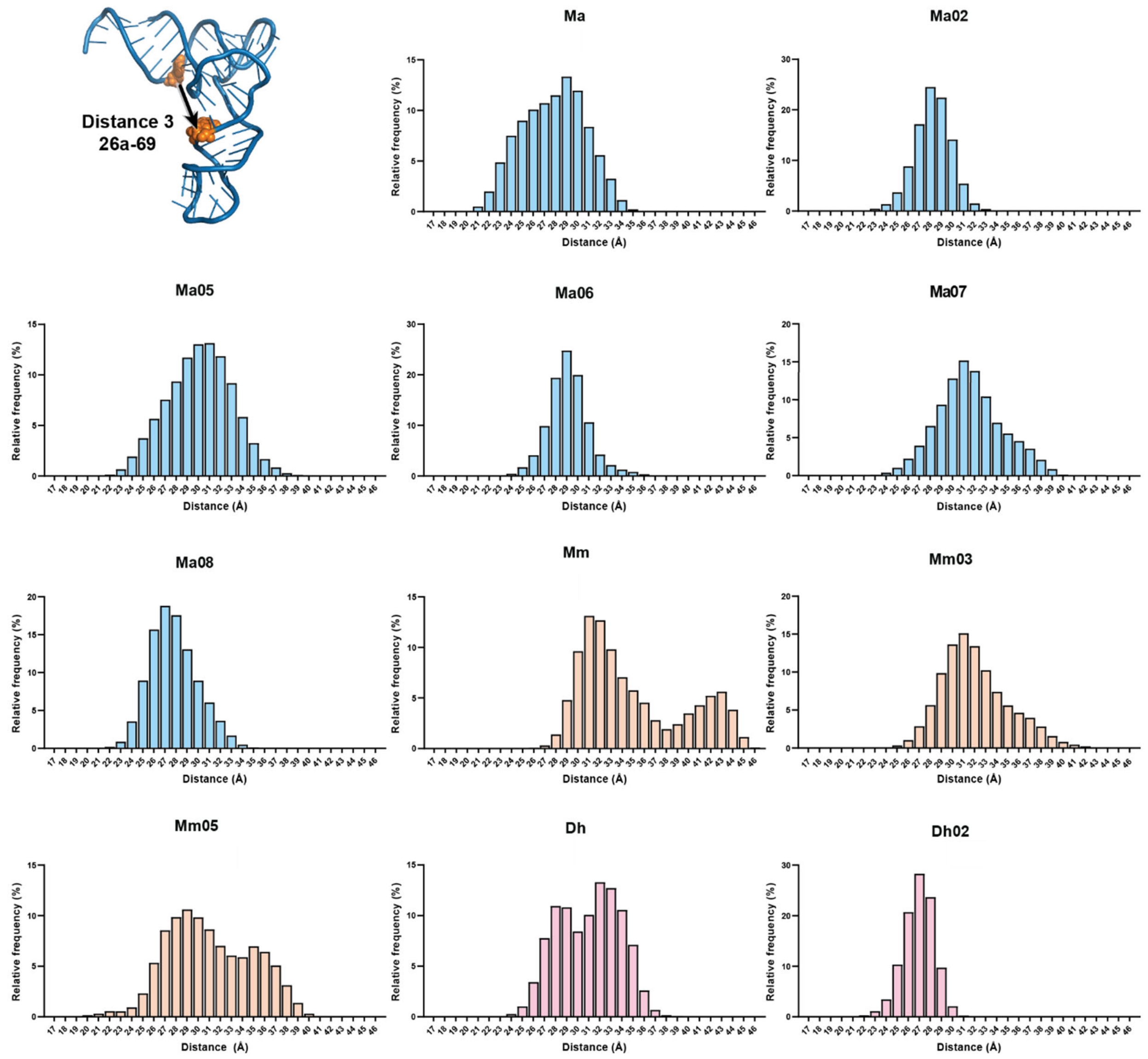

Supplementary Figure S7. Distance distribution of the simulated tRNA^{Pyl} variants from nucleotide 26a-69 (distance 3). tRNA^{Pyl} variants that are well-recognized by MaPylRS have a narrow distribution centered around 27 Å.
